# Drivers and constraints to environmental sustainability in UK-based biobanking: balancing resource efficiency and future value

**DOI:** 10.1186/s12910-023-00908-x

**Published:** 2023-06-01

**Authors:** Gabrielle Samuel, Jessica M Sims

**Affiliations:** 1 Department of Global Health and Social Medicine, King’s College London, London, UK; 2 Division of Surgery and Interventional Science, University College London, London, UK

**Keywords:** Biobank, Biobanking, Biosamples, Bioresource, Health, Environmental impacts, Sustainability, Value, Values, Ethics

## Abstract

**Background:**

Biobanks are a key aspect of healthcare research; they enable access to a wide range of heterogenous samples and data, as well as saving individual researchers time and funds on the collection, storage and/or curation of such resources. However, biobanks are also associated with impacts associated with a depletion of natural resources (energy, water etc.) production of toxic chemicals during manufacturing of laboratory equipment, and effects on biodiversity. We wanted to better understand the biobanking sector in the UK as a first step to assessing the environmental impacts of UK biobanking.

**Methods:**

We explored the sample storage infrastructure and environmental sustainability practices at a number of UK biobanks through a mixed methods quantitative and qualitative approach, including information gathering on an online platform, and eight in-depth interviews.

**Results:**

Environmental sustainability was deprioritised behind biobanks’ financial sustainability practices. Nevertheless, both often aligned in practice. However, there was a tendency towards underutilisation of stored samples, the avoidance of centralisation, and providing accessibility to biosamples, and this conflicted with valuing sustainability goals. This related to notions of individualised and competitive biobanking culture. Furthermore, the study raised how value attachments to biosamples overshadows needs for both financial and environmental sustainability concerns.

**Conclusions:**

We need to move away from individualised and competitive biobanking cultures towards a realisation that the health of the publics and patients should be first and foremost. We need to ensure the use of biosamples, ahead of their storage (‘smart attachments’), align with environmental sustainability goals and participants’ donation wishes for biosample use.

**Supplementary Information:**

The online version contains supplementary material available at 10.1186/s12910-023-00908-x.

## Introduction

Biobanks are a key aspect of healthcare research; they enable access to a wide range of heterogenous samples and data, as well as saving individual researchers time and funds on the collection, storage and/or curation of such resources. Over the past few decades, research funding agencies have driven the development of an increasing number of biobanks globally. This funding has supported the establishment of new (national) facilities (for example, UK Biobank, CanPath in Canada, All of Us in the United States). At the same time, biobanks remain heterogeneous. For example, in the UK, biobanks may be housed in universities or in National Health Service (NHS) hospitals, vary in size, and can be population or disease-based. Furthermore, within the UK there are many types of sample collections that can be accessed via application for further research even though they may not use the term ‘biobank’. There are also a range of stand-alone collections that are sometimes managed by biobanks. For example, within England, ‘Research Tissue Banks’ are centralised facilities which obtain ethical approval to facilitate programmes of research [1]. In Scotland, there is a network of ‘biorepositories’ that release samples via a single application system [2]. The UK also has a strong history of ‘cohort studies’ which store collected biosamples and data and can support the collection of new biosamples and data from the cohort.

This array of terminology is compounded by a lack of national surveillance and means that there is no definitive number of sample providers in the UK. Though we can estimate: in 2018, there were a reported 298 Research Tissue Banks in the UK [3], and the UK Clinical Research Collaboration (UKCRC) Tissue Directory has 289 registrants which include biobanks, biorepositories, bioresources, clinical trials, cohort studies, and Research Tissue Banks. Registration is voluntary, however, and does not include all the possible sample providers in the UK.

This lack of surveillance in biobanking means there is little knowledge about the environmental impact of biobanking as a sector (environmental sustainability). Broadly, the environmental impacts of biobanking include those associated with the laboratory waste (e.g. single use plastic) and/or hazardous chemicals generated in research laboratories where biosamples are prepared for storage and, in some biobanks, also analysed. Biobanks are also associated with impacts associated with a depletion of natural resources, production of toxic chemicals during manufacturing of laboratory equipment, and effects on biodiversity. A recent study published in the Lancet calculated the supply chain for research activities to have the biggest impact on biodiversity at Oxford University (UK) —greater than impacts that come from international flights, the university’s consumption of electricity, or the university’s use of construction materials [4]. Globally, and because of climate change and its associated health and environmental hazards, carbon emissions have gained particular attention as a specific environmental impact that needs consideration. On average, a research lab uses approximately four to five times more energy than similarly-sized commercial space [5]. Ultra-low temperature sample storage approaches (ultra-low temperature (ULT) freezers (-70/-80 degrees) and liquid nitrogen (LN2)) that house biosamples are perceived to be a key contributor of biobanks’ carbon emissions (and potentially other environmental impacts, though less is known about this). The lower the temperature the more energy required, and minus 80-degree freezers need 65× more energy than a regular household freezer (equating to that of an average UK household) [6]. (There have been calls for biobanks to raise the temperature of their freezers from − 80 to -70 degrees [7]). Furthermore, this energy usage significantly increases if freezers are not defrosted regularly [8]. These freezers also need to be housed in temperature-controlled rooms, which require energy to be maintained, and need to be replaced on a regular basis (every decade or so), which leads to waste.

There is an increasing moral imperative for health research to consider its own environmental impacts [9]. In fact, healthcare research has a special interest in addressing environmental impacts, not only as a matter of international priority, but also as a commitment to health [10]. In the biobanking arena, Samuel and colleagues have proposed a sustainability framework that includes the three pillars of financial, environmental, and social sustainability [9]. In this framework, *environmental* sustainability focuses on questions relating to the protection of the natural environment; *social* sustainability focuses on social justice issues, and ensuring the equal allocation of burdens, risks, benefits, and opportunities that may come from development within all societies; and *financial* sustainability includes *operatorial* sustainability and *social acceptability* [9] (also see [11]).

At present, literature on how current biobank practices pertain to questions of environmental sustainability remains scant, despite the growing focus on laboratory sustainability more generally (see, for example, the Laboratory Efficiency Assessment Framework (LEAF) [12] and mygreenlab [13]; also see [14]). We wanted to better understand the biobanking sector in the UK as a first step to assessing the environmental impacts of UK biobanking. To do this, we explored the sample storage infrastructure and environmental sustainability practices at a number of UK biobanks.

This is a preliminary study, and larger, quantitative studies will be needed to better understand the extent and generalisablity of our findings.

## Methods

We explored the sample storage infrastructure and environmental sustainability practices at a number of UK biobanks through a mixed methods quantitative and qualitative approach, including information gathering on an online platform (survey), and eight in-depth interviews.

### Survey

UK biobanks were invited through advertisement to answer questions on an anonymous Microsoft form about their biobank sample infrastructure and environmental practices. Advertisement was via the UKCRC Tissue Directory and Coordination Centre e-newsletter (n = 539 contacts), UK Biobanking Showcase conference delegates (n = 246), and to stand-alone collections and biobanks registered on the UKCRC Tissue Directory (n = 265 contacts). Questions asked about biobank type, institutional housing, funding arrangements, sample preservation and digital infrastructures,^1^ as well as practices associated with environmental sustainability. The question schedule is provided as supplementary material. Questions were reviewed by two independent scholars and piloted on one biobank before being disseminated more widely. Questions were preceded by a form providing information about the project (including a link to an online copy of the participant information sheet), and respondents who were happy to take part in a follow-up interview were directed to input contact details.

### Interviews

Eight respondents agreed to be interviewed and were contacted to provide more information on the project, a consent form, and arrange a time for interview. Interviews were online between January-June 2022, digitally recorded, and explored information about their biobank’s sample and data storage infrastructure (number of freezers, place where freezers are), awareness of environmental costs associated with biobanking, and questions associated with any environmental sustainability practices (do they have practices and what are they? What are their views on, and experiences of, any practices?). Views on responsibilities associated with these practices were also explored. Interviews lasted between 30 and 50 min (mean = 43 min). Interviews were transcribed and analysed by GS using inductive thematic analysis [15]. The interview transcripts were read and re-read and key themes were noted in a memo. GS then coded the data using descriptive codes. These codes were analysed to identify relationships and to develop the high-level thematic concepts that emerged in these findings: sustainability (financial and environmental), quality and value.

### Limitations

We cannot calculate the exact response rate because, as discussed above, the survey was circulated both indirectly via the UKCRC Tissue Directory and Coordination Centre e-newsletter (n = 539 contacts) and the UK Biobanking Showcase conference delegates (n = 246), as well as directly through stand-alone collections and biobanks registered on the UKCRC Tissue Directory (n = 265 contacts). Furthermore, these are overlapping, with some biobanks receiving the newsletter, attending the conference and/or receiving a direct email from us. Nevertheless, the number of biobanks that completed the Microsoft form (n = 22) meant that the response rate was below 10%, which is typically low for this type of study (generally around 20%). This was perhaps because we asked questions that respondents felt unable to answer (questions about electricity, number of freezers, data storage, etc.). Alternatively (or additionally), previous studies have reported health researchers to have a low interest in sustainability issues [14]. The low number may be a source of bias because it is likely that those who responded may have been more environmentally conscious than those who did not. A larger study will be needed to ascertain the extent and generalisablity of our findings. Eight follow up interviews were conducted. All interviewees managed a biobank, of which five of these biobanks were based at a university; n = 2 were housed in a university hospital, and n = 1 was within the UK National Health Service. Three of the biobanks were prospective biobanks. The number of samples (aliquots) held by the biobanks ranged from in the thousands, to the millions.

### Findings

Twenty two biobanks completed the online form between December 2021 and May 2022. Respondents were from England (n = 18), two from Wales, one from Scotland and one Northern Ireland. Fourteen institutions were housed in academic institutions, with the rest being National Health Service (NHS) based facilities, bar one, which was a registered charity. The current number and distribution of biobanks in the UK is currently unknown, so we are unsure how this compares to the population of biobanks nationally. Most biobanks (n = 14) were over 10 years old (n = 19 were over 5 years old), with n = 7 housing samples that were older than the biobank. The majority of the biobank representatives who completed the form were holding more than 10,000 biosamples (n = 16); n = 5 biobanks were prospective biobanks (only holding samples for a short time).

While considerations associated with biobanking’s financial sustainability were not the focus of this study, this emerged as an important aspect of interviewees’ discussions, and was often prioritised over concerns about environmental sustainability. Having said that, at the same time, promotion of financial sustainability often had a positive knock-on effect for environmental sustainability goals. Nevertheless, interviewees also reported a biobanking culture that conflicted with achieving both environmental *and* financial sustainability goals: value attachments to the biosamples factored prominently during biobanking decision-making, and often overshadowed both aspects of sustainability. We describe this in more detail below.

#### Financial sustainability

Interviewees required resources to conduct a range of activities to maintain the operations of their biobank. Nearly all interviewees reflected on the amount of time, effort, and money it took to run a biobank on a day-to-day basis:

*people think it’s easy. It’s like, what do you do? You just shove boxes in freezers? No, we don’t. Every single sample has to be given a unique ID and checked into a unique location and then checked out again* (interviewee 8).

As such, interviewees placed financial sustainability–and, in particular, the need for resources and funding to support the biobank’s activities –high on their agenda: *‘if you’re not financially sustainable there’s no point because nobody would actually have a job’* (interviewee 7).

Securing financial resources was difficult: n = 12 of the online respondents had at least some aspect of project-based funding, and for n = 10, this was time-bound. Interviewee 6 reflected on the need to continually apply for funds: ‘*our main agenda is sustaining the biobank, having enough money to keep running…because of the way we are…grant-funded’.* Interviewee 8 explained how their biobank was established to be self-funding, but its actual cost was not realised at the time, and their current financial structure required subsidisation from the university:

*the idea was that we would be self-funding. now what the university didn’t realise at the beginning-and has in the last couple of years woken up to with an almighty shock-is exactly why you don’t get core funding nationally for biobanks -because it’s bleeding expensive…way more than people in academia [will pay]. So the university has to subsidise a bit and it doesn’t like doing it, but it’s found that it’s got to…to get money into the university….*

Other biobanks had no subsidies, or at least spoke about how they had lost some of their funding: ‘*we used to have a technician solely dedicated to this [biobank sample handling], but we lost the funding. So now we’re a bit thin on the ground’* (interviewee 1). This lack of resources meant that biobanks had to focus on being operationally streamlined as much as possible: *‘can we look at the best staff to do things, so that we can make sure we’re running as efficiently as possible…from a processing human point of view* (interviewee 6). It also meant that social sustainability goals, such as an ethic of diversity and/or patients being included in biobank processes, were de-prioritised:

*we don’t have enough funding to target enough people from a different background….we’re aware of it, we don’t have I feel significant funding to do something about it…[moreover]…we’re asking [participants] to review… applications, to be involved, but we’re not able to compensate them in a way I feel that we should be* (interviewee 3).

Interviewee 6 explained, *‘it gets to a stage, if you cut any more, we might as well pack up and go home’*. Interviewee 3 was considering exit strategies for the “worst case scenario”: ‘*the UK biobanks at the moment…are in a dire state in terms of financial funding.[.].we’re having to discuss, you know, if the worst case scenario happened, what would be our exit strategy.[.]. we struggle year-on-year, everyone does…’*.

This lack of financial funding frustrated several interviewees, who spoke about a lack of funding foresight. For example, an online respondent reported how research funding bodies had allowed the collection of samples in numbers that far exceeded the usage needed, with a lack of consideration for the *‘downstream costs (financial and environmental) associated with long-term management and storage’*. Interviewee 8 reflected on how institutions receiving funding for biobank establishment had diverted resources away from the development of much needed biobank infrastructures and facilities.

### Environmental sustainability

While nearly half (n = 10) of the online respondents noted a lack of best practices associated with the environmental impacts of their biobanks, both online form and interview findings suggested that environmental sustainability was increasingly being considered in the biobanking arena (‘*I’ll hold my hand up, it’s not high on our agenda…but it is something that we are clearly starting to think more about’* (interviewee 6)), as part of a broader effort associated with the sustainability of research. For example, interviewee 5 noted how environmental sustainability was being raised in broader research and development agendas: *‘[our] keynote speaker at our last R&D open day [spoke about environmental sustainability]. To say that it was a sombre experience would be an understatement…’*. Online respondents reduced environmental impacts by minimising electricity use (n = 12); plastic (n = 9) and non-plastic (n = 9) waste; and/or considering the environmental costs of transport and manufacturing (n = 7). Some online respondents reported using research sustainability accreditation systems such as mygreenlab and LEAF (n = 2), or had other certifications of their environmentally sustainable research practices, such as those associated with ISO environmental Management Systems. One biobank was participating in an international freezer challenge to keep energy costs low. Other efforts included pushing suppliers to offer more sustainable products (n = 1) and turning lights/computers of at night.

There was a particular awareness amongst interviewees about the environmental impacts of freezers (‘*I sit on the college’s faculty sustainability committee now, and that’s around freezers’* (interviewee 2)). All online respondents had ULT − 80^o^C freezers (one respondent did not know if their biobank had these freezers; freezer numbers ranged from n = 1–80), and several online respondents had freezers below − 80 degrees or liquid nitrogen storage, with temperatures ranging from minus 150-196^o^C. Freezers were often housed in temperature-controlled rooms (n = 17; mostly between 1–5 rooms (n = 14)) that were between 15-20^o^ C (nearly all between 16-22^o^C; discrepancies likely to be related to the size of room and number of freezers. Sometimes air conditioning units do not work properly). Though only two respondents knew the electricity costs for their freezer rooms, both estimating them to be approximately £9000/year (no information on wattage provided; however, one of these biobanks housed 12 freezers, the other housed 13 freezers). Interviewees spoke about a range of actions they took to ensure the environmental impacts of freezers were kept to a minimum. This included minimal opening of freezer doors, cleaning freezer filters regularly and using energy efficient machine models where possible. Interviewee 3 explained:

*we try to do things like make sure our freezers are replaced regularly with the more energy efficient models, we have cleaning and defrosting programmes. We have even been looking at things like how regularly we clean out the air filters and clean our freezer room to make sure that the freezers aren’t having to work harder….*

### Environmental sustainability as a by-product of financial sustainability: alignment of goals

While environmental sustainability was viewed by interviewees (and seen in the online data) as an increasingly important issue for biobanks to consider, any environmental agenda was generally perceived to sit behind financial priorities. Nevertheless, interviewees gave examples of environmental sustainability efforts seemingly being an accidental by-product of financial considerations. Interviewee 6 described their financial considerations about freezer performance as having environmental impacts too: ‘*maybe the environmental impact wasn’t necessarily the driver for it….the newer models maybe just happened to have a better [energy rating]’.* (We note that this comment assumes that buying more efficient freezers is the most environmentally sustainable choice. It might be more environmentally sustainable to delay buying a new freezer depending on the energy consumption associated with the manufacture of a freezer compared to use. There will likely be ‘pinch point’ when it becomes more environmentally sustainable to invest in a new freezer.) In a different context, interviewee 1 explained how the biobank’s director wanted to refrain from new freezer purchases (an environmental cost) because of the financial cost:

*we could have an additional one or two freezers. But the [director has said] “no, we don’t want to extend any more, we should be able to be sustainable as we are” So I’m not sure it comes primarily from let’s be more sustainable, to be honest. But it’s also obviously a matter of cost….*

Other aspects of biobank operations were more *explicitly* aligned financially and environmentally. For example, interviewees repeatedly emphasised the financial benefits associated with the need for biobanks to ensure stored biosamples were being used, because this not only brought income to the facility, but also reduced the financial and environmental costs associated with any indefinite freezer storage. Importantly for a number of interviewees, it also related to ensuring the purpose of the biobank and desires of participants (i.e., the desire to have their samples used for research) were being met. Interviewee 7 explained:

*the most important thing is making sure that you’re using samples, that you’re not collecting samples that will never get used. There needs to be a purpose for what you’re doing. And that works for financial sustainability as well as environmental sustainability.*

To improve sample usage, interviewees described the need to improve the visibility of biobanks to researchers; others described sharing the details of other biobanks with researchers if they were unable to assist with researcher requests for samples. This sharing mentality was important, explained interviewee 7, because it reduces the chance of wastefully re-collecting a set of samples that had already been collected: *‘if somebody’s got a set of samples from people with a particular disease [and you know about it], why would you bother to go to the extent of setting up the whole new collection yourself….’.*

Finally, biobank centralisation within a particular institution was perceived to be financially and environmentally aligned. As some interviewees explained, this was because locally managed freezers were perceived to be poorly managed compared to a more efficient centralised facility:

*we’ve got freezers all over the place, tucked in completely inappropriate areas..[that are very poorly managed]….I think a lot of them [researchers] will be happy to get rid of some of the freezers [to a more efficient off site facility]…but they want to know that any facility they use is cheap to use, and that they can access things when they want, and the things don’t get lost* (interviewee 2).

In fact, a number of biobanks which responded online already stored their biosamples centrally (n = 14 within an institution or purpose-built facility), though n = 3 respondents reported that their ULT − 80^o^C freezers were housed locally in the research laboratory, and another five respondents stated that their ULT − 80^o^C freezers were located in a number of places, including the laboratory. Interviewee 1 reflected on their own and other similar biobanks in their institution, which were not yet centralised institutionally, and specifically how each biobank was unsustainably buying their own equipment and products: ‘*we still have a few independent biobanks, and we operate in a slightly different way.[.].and I don’t think that people who are buying [for the biobanks], well every group will buy their own things’*.

### Biobanking culture

Despite interviewees’ endorsements of financial and environmental sustainability, they stressed that many biobanks – sometimes themselves – struggled with adopting practices to address these priorities because of a conflicting biobank culture. The mentality associated with the need to collect and store (rather than use) biosamples was one example:

*I don’t want to just keep asking for more and more freezers. The whole idea of biobank is we give it away as well….Too many biobanks, and I’m probably guilty of that as well, we’re just collecting and we keep wanting more and more storage facilities. And I’m not sure that’s right but that’s the balance we have at the moment* (interviewee 2).

This stockpiling mentality was perceived to be shared by researchers who housed their own biosamples – though perhaps for different reasons. For example, interviewee 8 described situations in which researchers requested that their institutional biobank service collect biosamples because of a perceived future need, even though those samples had remained unused:

*[researchers say] I don’t have the money and I don’t need them [samples] right now [but they will be valuable] so just make the collection and then I’ll get everything out. So we did a lot of processing…and of the 18,500 samples that were collected, only 100 have been asked for.*

Storing biosamples for no specified future use has financial and environmental cost implications. These could potentially be mitigated by making samples visible to other researchers, perhaps through a centralised system of storage. However, interviewees also described a culture in which researchers house and keep their own smaller collections of samples rather than centralising them in a biobank and/or sharing information about them with others (when consent agreements allow). This was perceived to be related to aspects of control (by those who had collected the samples) and trust (in those who would potentially use and/or store them). It was also perceived to be attached to the time and effort associated with setting up research studies and collecting the samples:

*it takes a lot of time and effort to set up, to get some funding to collect and to have somebody processing samples, get them, retrieve them, etc….obviously, people are quite protective of the sample that they’ve taken years to collect….people may fear that they will lose some of that control* (interviewee 1).

At the same time, as the two extracts below illustrate, interviewee 5 stressed that having a central facility was not a technological fix to financial and/or environmental concerns because with more space and less control, researchers could store their samples in the biobank indefinitely, even if they changed institutions (or bring them from other institutions, and then leave them when they moved on):

*we received three − 80 freezers, full of samples from [one university], we fully inventoried them, we reopened the study, he moved on. He left two full freezers here*;

*all of our local PIs [principle investigators] think that they can leave samples with us for an indefinite period of time…So we gradually accrue samples for studies and then people move on and then not tell us that they’ve moved on, or studies close and they don’t tell us that they’ve closed, so we end up looking after the samples rather longer than we would hope to*.

Though, there was a sense, at least from some interviewees, that biobanking culture was (very) slowly changing, and that there was an increasing realisation that biobanks—and the biosamples collected by researchers—were established ‘for the health of people’ rather than for researchers themselves (or biobanks), and that the value of a biosample comes from its use rather than from its storage:

*historically the, what’s the word, the atmosphere within tissue banks has been kind of “no, that’s my tissue, I’m going to keep it, I’m going to use it, and it’s not to share”. But it seems to be changing now where people are kind of thinking “you scratch my back, I’ll scratch yours”, you know, that we’re not doing it for us, we’re doing it for the health of people, the general public* (interviewee 4).

### The preservation of valuable biosamples at any cost

Interviewees spoke about the need to carefully balance biosample quality preservation versus environmental impacts. Interviewee 6 described decisions about re-arranging freezers for storage efficiency versus the impact of freeze thaw cycles on the quality of samples:

w*e will occasionally have a move round of samples to make sure that we’re using things most efficiently. Again, the balance of that is, well, to be able to do that you have to take them out of the freezer, to be able to log them….it’s a little bit of a balance between having the samples out of the freezer, even for a short period of time…But yet we do try.*

Though more often than not, an environmental agenda was de-prioritised behind the preservation of biosample quality: ‘*it’s not my first decision [about environmental sustainability], my decision is around the quality of the material and whether it has research value’* (interviewee 2). In many instances these choices related to regulatory requirements. Interviewee 8 explained that such requirements were necessary because of the need for biobanks to store long-term. Choices included the use of virgin plastics so that specimen quality was not compromised (*‘it’s got to be virgin plastic’* (interviewee 8));^2^ the need to buy specific freezers (‘*[we] don’t choose to use the most energy efficient model because it doesn’t provide the highest level of safety’* (interviewee 3); having back up freezers (‘*for every five freezers, you’ve gotta have one as backup. Where yes, you are paying electricity to maintain…nothing, because the HTA requires you to have contingency’* (interviewee 8)); and keeping freezers at -80 degrees Celsius rather than − 70 because samples degrade increasingly at higher temperatures:

*in terms of sustainability, we’re slightly…limited…We have to adhere to the Human Tissue Act and meet our ethical requirements around protection of the tissue… sometimes I feel the two can conflict slightly because obviously we need to go legislation first* (interviewee 3).

However, these choices often went beyond regulatory compliance, and often beyond a financial versus environmental trade off: as custodians of a biobank, interviewees viewed the need to maintain biosample quality as the only morally appropriate option. This was tied to a perceived value attached to each biosample, emphasised using language such as ‘precious’ (interviewee 4) and ‘irreplaceability’ (interviewee 3). For example, in response to the possibility of raising the temperatures of their biobank ULT − 80^o^C freezers, interviewee 3 remarked: ‘*to be honest it probably would be fine, but I’m not willing to stake an* *entire irreplaceable tissue* *collection on it’.* This irreplaceability was connected to the effort that had been put into securing these samples, as well as the participants who had donated them: ‘*it’s irreplaceable, it’s people whose families I’ve spoken to, people I’ve known over the years’* (interviewee 3).

The importance placed on this perceived value of biosamples emerged in other instances, for example in considerations associated with discarding samples. Here, some (though not all) interviewees reflected on the need to discard biosamples that were not, and had not been, used for a long time to ensure costs of storage were reduced (*‘I’m mindful of the fact that we’ve got these things stored and they will cost a lot of money to manage and keep going’* (interviewee 2)). Often this balance erred on the side of caution because of strong regulatory requirements surrounding the discarding of samples. For example, interviewee 6 explained how the biobank had samples that might never be used, they only destroyed samples if issues arose with sample integrity. However, this caution was also because of the perceived ‘potential future research’ (interviewee 8) value of tied to the biosamples: ‘*people are so scared of getting rid of it [sample] because they’re gatekeeping this precious tissue [that might have future value]’* (interviewee 4)). This led to an overall reluctance to discard samples. Interviewee 2 provided an example of a project that collected too many vials of sample per participant, but for which a request to discard some unused vials to free up freezer space was still ongoing after years because ‘*no one was prepared to make that decision’* to discard biosamples with perceived value. This keeping of biosamples that had not been used for a long time, or were not likely to be used, frustrated some interviewees, who described unnecessary storage of biosamples as ‘wholeheartedly pointless’:

*they [the institution visited] had basements full of freezers…..There must have been, I don’t know, 40 that were just sat there that…hadn’t been used in a long time or hadn’t been opened. Because even the guy, who is a friend of mine, was saying it’s ridiculous. “I come down here and write the numbers of the fridge down here and it hasn’t been opened in three and a half months” It just seemed wholeheartedly pointless[(from an environmental and financial point of view].*

## Discussion

Our findings show how awareness of the adverse environmental impacts of biobanking is increasing, and those working in the field are adopting more environmentally sustainable practices (turning off lights, trying not to open freezer doors, considering aspects of waste). This seems to be spurred by the broader awareness of environmental impacts in (health) research fields more broadly (for example, see [5, 16–20]). Nevertheless, concerns associated with environmental sustainability were often overshadowed by anxieties related to maintaining the financial sustainability of biobanks-the latter being connected to previously published concerns about precarious public funding that did not allow formal planning for long-term stewardship of biobanks, nor the operation of biobanks beyond critical staffing levels [21] (also see [11, 22–25]). Focussing on financial sustainability was not necessarily problematic when aligned with environmental sustainability practices goals, such that practices that promoted both were intricately tied. However, interviewees did suggest the presence of a research culture of individualised control that could conflict with both of these goals, such as by not wanting to centralise and/or provide accessibility to biosamples. They also pointed to a tension between sustainability goals and the value biobankers and researchers often attach to biosamples.

Research practices that prioritise individualised control of biosamples have been noted by others, where they are linked to difficulties with researchers being able to find biosamples with good quality data, and obtain access to them from non-local sources [26–28]. This individualised mentality to control has been shaped, at least in part, by the performative pressures and competitiveness associated with ‘the neoliberal cascade’ of marketised academia [29, 30]. As Giroux (2004) emphasises, in such a neoliberal culture academics are ‘*entrepreneurs who view the future as an investment opportunity and research as a strategic career move rather than as a civic and collective effort to improve the public good*’ [31](p. 232). Furthermore, the way in which academic research is evaluated at the individual level has created a culture that intimates ownership and a sense that academic outputs are an individual’s property [32]. It might be more helpful and profitable to think of ideas not as academic capital but as public interest goods [32]. Notions of solidarity and justice are good ethical principles to draw on here, and are used to formulate increasing moves to drive open science more broadly across the research sector by adhering to the FAIR principles (findable, accessible, interoperable and reusable) [33–36].

At the same time, our findings suggested that much of the desire for researchers to hold onto their biosamples also emerged from a sense of attachment associated with the amount of work invested into collecting the biosamples [37, 38]. Attachments between individuals and non-living entities have been reported previously [39], and can be a result of both (or either) emotional connection and/or cognition [39]. At the same time, it is vital to distinguish whether such attachments are ‘smart attachments’ (cf. smart trust, O’Neil [40], whereby the reason to have an attachment with an object (e.g., a biosample) is both worthy and justified. Here, it could be argued that an attachment to a biosample that was associated with effort invested is not ‘smart’ because it would prioritise biosample storage rather than use. In fact, an international survey of biobanks have reported utilisation rates of 10% or lower [41]. Collecting samples that are not utilised and without market need has been emphasised to be ‘*financially unsustainable and socially reckless’* [41]. A more appropriate attachment—also identified in our findings—might be one developed between biobankers and participants who have donated biosamples. This scenario facilitates and supports biosample *use* because it is the attachment to the participant (and the participant’s desires to have their sample used for research purposes [42]) that is important here, rather than that associated with the biosample.

Finally, attachments to the biosamples were linked to perceptions associated with the potential future value of the biosamples for health research purposes. Much literature has explored value in biobanking, and in particular, how biosamples (and/or data) are constructed into commodities (or assets [43]) in the bioeconomy (see, for example, [44, 45]). Here values become socially constructed [46]—tied to a discourse of potential expectations, promises and hype [47–50]—which creates a socio-technical imaginary that views health as fixable by technocentric means (innovative and technology-driven health research) rather than via considerations associated with the social determinants of health [51–53]. This does not negate biobanks as an important driver in advancing healthcare research and providing health value [54], and some of our interviewees spoke about health research that had been underpinned by the use of their biobank’s samples. Nevertheless, it does leave tricky questions regarding how much emphasis to place on the potential value of the biosamples, and what this should mean in terms of current practices. For example, in the current climate emergency [55, 56], we must support biobanks, but we must think about how this can be done in a way that is considerate to the environment (and the detrimental health consequences that are and will come from this). Here and in other literatures, we see how perceiving biosamples through the lens of future potential health research value could drive the collection of increasing samples with little constraint. In our findings, we can see how the value attached to biosamples has resulted in researchers being hesitant to make their collections visible to other researchers and/or place their samples in centralised collections for further use. This has environmental implications because it means that biosamples are not used to their full potential; it also means that the duplication of biosample collections may occur.

## Conclusion

In conclusion, the biobanking sector seemed to be increasingly concerned about its environmental impacts. While these concerns were often overshadowed by the need to maintain financial sustainability, the two (economic and environmental values) often aligned in practice. Nevertheless, there was a research culture that avoided centralisation and/or providing accessibility to biosamples, as well as a desire to hold onto biosamples. We need to move away from this individualised and competitive culture towards a realisation that the health of the publics and patients should be first and foremost. We need to ensure the use of biosamples, ahead of their storage (‘smart attachments’), align with environmental sustainability goals and participants’ donation wishes for biosample use.

### Recommendations

To ensure that biobanking moves towards greater environmental sustainability, we need to consider and adopt practices which facilitates their efficient use. Collected biosamples must be well characterised and linked to health data. It is this data that adds to the research value of biosamples, especially in cases when they are collected without a specific demand or application. Poor quality data prevents biosamples from being made usefully visible and, in turn, less relevant to future use [28]. These collections would also be easier to track during centralisation, release for use, and destruction, thereby preventing the need for increasing storage facilities—an environmental goal—as well as aiding with forecasting costs for financial sustainability. Furthermore, biobank collections should only be collected for a specific purpose (and there should be a demand for them). We should ensure that the infrastructure is established before funding and the maintenance funding is secured to ensure any collection of samples is not wasted from lacking the resources for rerelease.

## Electronic supplementary material

Below is the link to the electronic supplementary material.

**Additional File**: Survey of sample & data storage practices: biobanks & health research data repositories

## Data Availability

The datasets analysed during the current study are available from the corresponding author on request.

## Footnotes

1 Findings associated with digital infrastructures are discussed elsewhere.

2 The need to use single use plastics to avoid contamination has come under increasing scrutiny, with a wave of activity beginning to explore the usefulness of multiuse equipment.
