# Supplementary material for: Drivers and constraints to environmental sustainability in UK-based biobanking: balancing resource efficiency and future value
Source: BMC Med Ethics. 2023 Jun 1;24:36. doi: 10.1186/s12910-023-00908-x (PMC10236775; doi:10.1186/s12910-023-00908-x)
Supplement: Supplementary file 1 — Additional File: Survey of sample & data storage practices: biobanks & health research data repositories [file 12910_2023_908_MOESM1_ESM.pdf]

Survey of sample & data storage practices: biobanks & health research data repositories

We would like to invite you to participate in this survey. Before you decide whether you want to, it is important to understand why the survey is being done and what your participation will involve.

This survey aims to capture information about the environmental footprint of the UK biobanking/health data repository sector. It will do this by surveying information on sample storage, data storage and lab practices at your biobank/data repository. The aim is to explore possibilities for decreasing the environmental footprint of the sector, while maintaining high quality practices. To do so, it is vital that we understand current biobank/data repository practices. It may be that your organisation already has environmental sustainability related practices underway. We want to learn about these so we can create best practices for others. The survey has three parts:

- demographic questions
- (if relevant) your biobank's sample size, number of freezers, and lab-based sustainability practices
- (if relevant) how and where you store participant/patient data.

All required survey questions are tick box or one-word answers (with options for more information if you choose). The survey should take about 3/4 minutes, though each section of the survey may require expertise/knowledge from other members of your biobank/data repository/institution. If possible, please could you speak to these people, as we would like to gain as much information as possible so we can get an accurate picture of practices in the UK.

For more information about the survey, including why you have been invited, what will happen if you take part, data governance, and the possible risks/benefits of taking part [illegible]

1. I have read and understood the information provided and I agree to

1. I have read and understood the information provided and I agree to take part in this research project *

◯ Yes

2. Which country is your biobank or health research data repository based in? *

3. What type of institution houses your biobank or health research data repository? *

- ◯ Academic
- ◯ NHS
- ◯ Commercial small to medium enterprise (SME)
- ◯ Pharmaceutical company
- ◯ Registered company and registered charity

4. How long has your biobank or health research data repository been operating for?
If your biobank has been operating for less time than the length of time the samples have been stored, please answer in terms of the age of the biobank, and also tick the button*: The samples our biobank houses are older than the biobank* *

- ☐ Less than 2 years
- ☐ Between 2-5 years
- ☐ Between 5-10 years
- ☐ Over 10 years
- ☐ The samples our biobank houses are older than the biobank

5. Which mode of funding most applies to your biobank or health research data repository? *

- ☐ Operational or project-based funding via a charity
- ☐ Operational or project-based funding via a research council
- ☐ Operational or project-based funding via government body
- ☐ Income via independently funded projects
- ☐ Income via supporting commercial research
- ☐ Underwritten by host institution with expectation to cost recover
- ☐ Underwritten by host institution with no expectation to cost recover
- ☐ Other (please specify below)

6. Other: please specify

7. Is your funding time limited? *

- [ ] No
- [ ] Yes, for less than 2 more years
- [ ] Yes, for another 2-5 years
- [ ] Yes, for another 5-10 years
- [ ] Yes, for another 10+ years
- [ ] I don't know
- [ ] Not applicable

8. How are your samples and/or data accessed by researchers? *

- [ ] Application for access is granted by the PI of the project
- [ ] Application for access is reviewed by committee
- [ ] Application for access is through a research infrastructure or network

9. What is your role at your biobank or health research data repository?
*

- [ ] Researcher (including lab manager)
- [ ] Data scientist
- [ ] Governance (PI or other)
- [ ] Administration
- [ ] Other (please specify below)

10. Other: please specify

11. Roughly how many samples does your biobank or health research data repository contain? *

- [ ] 1-500
- [ ] 501-1000
- [ ] 1001-5000
- [ ] 5001-10,000
- [ ] over 10,000
- [ ] We collect prospective samples, or hold samples for a short time
- [ ] None, we are a data only repository
- [ ] I don’t know
- [ ] Other (please specify below)

12. Other: please specify

13. How many electrical freezers (-80 degrees Celsius) do you use to store your samples?
Please state approximately, or state 'n/a' if not applicable, or 'I don't know' if unsure

14. How many electrical freezers (between -80 & -140 degrees Celsius) do you use to store your samples?
Please state approximately, or state 'n/a' if not applicable, or 'I don't know' if unsure

15. How many liquid/vapour nitrogen freezers do you use to store your samples?
Please state approximately, or state 'n/a' if not applicable, or 'I don't know' if unsure

16. What temperature do you keep your liquid/vapour nitrogen freezers at?
Please state approximately, or state 'n/a' if not applicable

17. How many temperature-controlled rooms/air-conditioned rooms does your biobank use? *

- ◯ None
- ◯ 1-5 temperature-controlled rooms (room equivalent to roughly 16m2)
- ◯ 6-15 temperature-controlled rooms
- ◯ 15 plus temperature-controlled rooms

18. State temperature here

19. Where are the freezers that hold the stored samples?

	In the research group's laboratory In departmental laboratory/storage area	Purpose built facility within a department	Purpose built facility in a centralised location within the institution	Purpose built facility externally located from, but maintained by, the host institution	Purpose built facility externally located from, and hired by, the host institution	Combination of places (please provide detail below)
-80 degrees	○	○	○	○	○	○
-81 degrees and below	○	○	○	○	○	○
Liquid/vapour Nitrogen	○	○	○	○	○	○
n/a	○	○	○	○	○	○

20. If you answered '*Combination of places*' in the above question, please provide additional information here, otherwise skip this question

21. What is the amount of electricity and/or the energy cost of running any/all of the freezers?
*Please state approximately, leave blank if you don't know, or write **IL** if storage is at the institutional level*

22. Does your biobank laboratory have best practices (or any practices) associated with: (tick as many as appropriate) *

- ☐ Trying to minimise plastic waste
- ☐ Trying to minimise other non-plastic waste
- ☐ Considering transport and manufacturing environmental costs during procurement of devices/technologies?
- ☐ Trying to minimise electricity/energy use in your lab?
- ☐ Associating or follow the polices of sustainable lab practices (which ones?)
- ☐ None

23. Please provide as much information as possible regarding the above question (lab practices)

24. How much data is roughly stored as part of your biobank or health research data repository?
Data here refers to data that is associated with the individual from whom the sample came from, e.g., if any phenotypic, lifestyle or other health data was recorded; data from analyses of samples; or other data such as genomic, exome data etc. *

- ◯ Below 256 giga bytes [miss rest of the questions about data] (approx. 96,000 compressed photos; 1250 hours video; 50,000+ songs;)
- ◯ 257 giga bytes – 1 tera byte
- ◯ 1 tera byte – 50 tera bytes
- ◯ 51 tera bytes – 1 peta byte
- ◯ Exa byte range
- ◯ Zetta byte range
- ◯ I don’t know, but we are a biobank and only collect a small amount of data about our participants and don’t analyse the samples

25. Where is the data stored? *

- ◯ On a hard drive(s) in the laboratory/research group/department
- ◯ Centralised on a server within the institution that houses the biobank
- ◯ On an external server i.e., cloud (if so, please state which one below)
- ◯ Other (please specify below)

26. Other: please specify

27. What is the approximate energy consumption (in kW) of this data storage?
*Please state approximately, leave blank if you don't know, or write **IL** if your data is stored at the institutional level*

28. What type of energy is used to supply the computers that store your data? *

- ○ Mix of renewable and non-renewable energy, with over 50% renewable
- ○ Mix of renewable and non-renewable energy, with over 50% non-renewable
- ○ I don’t know because it would be at the institutional level
- ○ I don’t know
- ○ Other (please specify below)

29. Other: please specify

30. If external researchers access your data, how is this data accessed? *

- ◯ Through a cloud provider
- ◯ We/I send the data to the researchers online, and they download it
- ◯ We/I send the data to the researchers on a portable device, and they download it
- ◯ We/I do not permit external researchers to access to the data

31. Approximately how many times do you send data to researchers each year, and how much data is sent? (very roughly)?

32. Approximately how often do the hard drives on which your data is stored need replacing?
Please state approximately, or leave blank if you don't know

33. What happens to the old hard drives/servers when they are replaced? *

- ◯ Recycled
- ◯ Disposed
- ◯ Repurposed
- ◯ I don't know
- ◯ Data is stored on a centralised server, so I don't know

34. Do you or the researchers who use your data have specific processing requirements when analysing the data?
i.e., how much power/energy do you need from the computer to run data analysis – how fast do you need calculations to be etc

35. Does your biobank or health research data repository have any laboratory sustainability guidelines that you follow, and if so, what, briefly, do they require? *

- ◯ Yes
- ◯ No

36. If yes, which ones

37. Any other comments (including other environmental impacts that you consider in your decision-making, or which may worry you)

This content is neither created nor endorsed by Microsoft. The data you submit will be sent to the form owner.

Microsoft Forms
